# Dataset on the perceptions of ordinary people on the persistence of bribery practices in Nigeria

**DOI:** 10.1016/j.dib.2020.106616

**Published:** 2020-12-08

**Authors:** Abubakar Saddiq Sani, Abu Sufian B. Abu Bakar

**Affiliations:** a School of General Studies, Abubakar Tatari Ali Polytechic, P.M.B 0009 Jos Road, Bauchi, Nigeria; b School of Economics, Finance and Banking, College of Business, University Utara Malaysia, 06010 UUM Sintok, Kedah Darul Aman, Malaysia

**Keywords:** Anti-bribery measures, Demographic factors, Grassroots, Persistence, Societal acceptance, Socio-economic impacts

## Abstract

This dataset provides the perceptions of ordinary people on the relationship between societal acceptance of bribery, anti-bribery measures, socio-economic impacts of bribery, demographic factors and persistence of bribery practices. A multi-stage sampling technique was used to target the respondents. A total of 887 grassroots respondents participated, out of which 836 responses were used for quantitative data analyses. Statistical Packages for Social Sciences (SPSS) version 23 and SmartPLS3.0 were used to determine the reliability and validity of the data. The data is useful to the authorities in formulating strategies to deal decisively with the issues of bribery practices in Nigeria.

## Specifications Table

**Table:** 

Subject	Economics
Specific subject area	Economics of Crime
Type of data	Table, Figure
How data were acquired	A field survey was employed to collect the data
Data format	Raw, Analyzed
Parameters for data collection	Data on ordinary people's perception of persistence of bribery, societal acceptance, anti-bribery measures and socio-economic impacts of bribery were collected using survey questionnaire adopted from [1]
Description of data collection	A total of 1000 questionnaire were self-administered among the targeted population. After a thorough screening in SPSS version 23, 836 responses were used to analyze the data in SmartPLS3.0. Majority of the respondents were civil servants (*N* = 537, 64.2%), followed by private sector workers (*N* = 189, 22.6%) and self-employed individuals (*N* = 110, 13.2%). The questionnaire is provided as a supplementary material in this article.
Data source location	Abuja, the federal capital city of Nigeria
Data accessibility	The data are available in Mendeley Data: https://data.mendeley.com/datasets/962b4r3hb2

## Value of the Data

• The dataset provides the perception of the grassroots on the issues pertaining continued occurrences of bribery practices despite strategies, programs and policy measures put in place in Nigeria. The data is useful, as there exists a paucity of research on the perception of grassroots on bribery practices in Nigeria; the focus has always been on the perceptions of high profile individuals such as top business executives, lawyers, and international institutions among others.
• The dataset is also useful to the international and non-governmental organizations such as the United Nations (UN), World Bank, International Monetary Fund (IMF), Transparency International (TI), International Police (INTERPOL), Human Rights Watch, among others in their further researches on the impact of bribery practices especially in Nigeria and the world at large.
• The data are also valuable to local, state and federal government in Nigeria in dealing decisively with the issues of bribery practices as perceived by the grassroots. This is because the data provided an in-depth insight into the extent of societal acceptance and impacts of bribery practices in Nigeria.

## 1 Data Description

A survey questionnaire technique was employed to acquire the dataset. A 5 points Likert scale rating that range from ‘strongly disagree’ to ‘strongly agree’ was used in conducting the survey. The questionnaire consisted of five sections. Section A was designed to collect data on the demographics of the respondents: gender, age, marital status, family size, educational status, job status and annual income. Section B contains instruments meant to measure the dependent variable (perceptions on the persistence of bribery practices in Nigeria). Section C deals with instruments that measured respondents’ perception of societal acceptance of bribery in Nigeria. Section D comprises of instruments used to measure the respondents’ views on the impacts of anti-bribery measures in Nigeria. Finally, section E contains instruments employed to measures the respondents’ opinions on the socio-economic impacts of bribery practices in Nigeria. The questionnaire is provided as a supplementary material in this article. The individual response to the questionnaire are reported in the Mendeley dataset.

The first construct is the demographic factors of the respondents that represent the influence of the factors towards perceiving persistence of bribery practices in Nigeria where five items gender, marital status, family size, educational qualification and job status were included. The second construct is the persistence of bribery practices (dependent variable) that represents the respondents perception about widespread, continuity and permanency of bribery practices among others where ten items PBR_1 –PBR_10 were coded.

The third construct is societal acceptance of bribery practices that assesses the respondents’ opinion of justification, tolerance and openness of bribery practices in Nigeria where nine items SAC_1 – SAC_9 were coded. Anti-bribery measures is the fourth construct that assesses perception of the respondents towards the impacts of policies, institutions and programs put in place to reduce bribery practices in Nigeria where twelve items ABM_1-ABM_12 were coded.

Socio-economic impacts of persistence of bribery practices is the fifth construct that measures the socio-economic damages of persistence of bribery practices based on the perception of the respondents where fourteen items IMP_1-IMP_14 were coded. All the items are shown in Table 1.

**Table 1:** Constructs and items.

Constructs	Indicators	Labels (Codes)
Demographic Factors	Gender	GND
	Marital Status	MTS
	Family Size	FAS
	Educational Qualification	EDU
	Job Status	JOB
Persistence of Bribery Practices (PBR)	Bribery is widespread in Nigeria	PBR_1
	Bribery has become permanent in Nigeria	PBR_2
	Bribery has continued to occur in Nigeria despite combative policy measures put in place by the government	PBR_3
	Bribery is frequently offered to school officials in order to get services needed from educational institutions in Nigeria	PBR_4
	Bribery is frequently offered to medical officials in order to access medical services in Nigeria	PBR_5
	Bribery is frequently offered to government officials in order to get officials documents (such as birth certificate, tax clearance, national identity card, driver's license, voters card, and certificate of occupancy among others) in Nigeria	PBR_6
	Bribery is frequently offered to police officers in order to get their assistance or to avoid paying fine or arrest in Nigeria	PBR_7
	Bribery is frequently offered to court officials in order to get their assistance or to have judgment in one's favor in Nigeria	PBR_8
	Bribery is frequently offered in order to secure a job in Nigeria	PBR_9
	Bribery is the most important problem facing Nigeria today	PBR_10
Societal Acceptance of Bribery (SAC)	Bribery has become a way of life in Nigeria	SAC_1
	Bribery is justifiable in Nigeria	SAC_2
	Bribery is tolerated in Nigeria	SAC_3
	Ordinary government officials asking for bribes openly in Nigeria	SAC_4
	Ordinary people offering bribes openly in Nigeria	SAC_5
	Ordinary people do not report bribery practices to anti-bribery institutions in Nigeria	SAC_6
	Ordinary people have non-charlatan attitudes towards bribery practices in Nigeria	SAC_7
	Ordinary people aiding bribery transactions in Nigeria	SAC_8
	Everyone is involved in bribery in Nigeria	SAC_9
Anti-Bribery Measures (ABM)	Establishment of Code of Conduct Bureau (CCB) has reduced persistence of bribery in Nigeria.	ABM_1
	Establishment of Public Complaints Commission (PCC) has reduced persistence of bribery in Nigeria	ABM_2
	Establishment of National Orientation Agency (NOA) has reduced persistence of bribery in Nigeria	ABM_3
	War Against Indiscipline and Corruption (WAIC) has reduced persistence of bribery in Nigeria	ABM_4
	Establishment of Independent Corrupt Practices Related Offences Commission (ICPC) has reduced persistence of bribery in Nigeria	ABM_5
	Establishment of Economic and Financial Crimes Commission (EFCC) has reduced persistence of bribery in Nigeria	ABM_6
	Prosecution and conviction of offenders has reduced persistence of bribery in Nigeria	ABM_7
	Whistle blowing policy has reduced persistence of bribery in Nigeria	ABM_8
	Deterrence measures (such as enhancing integrity and transparency) have reduced Persistence of bribery in Nigeria	ABM_9
	Citizen advocacy measures have reduced persistence of bribery in Nigeria	ABM_10
	Civil Society anti-bribery campaigns have reduced persistence of bribery in Nigeria	ABM_11
	Targeting high profile bribery offenders has reduced persistence of bribery in Nigeria	ABM_12
	Socio-Economic Impacts of Bribery (IMP) When bribery is offered by ordinary people in Nigeria it is like a regressive tax	IMP_1
	Bribery discourages ordinary people from assessing public goods in Nigeria	IMP_2
	Bribery reduces the social welfare of ordinary people in Nigeria	IMP_3
	Bribery is responsible for erosion of ordinary people's trust in private and government institutions in Nigeria	IMP_4
	Bribery raises the cost of doing business in Nigeria	IMP_5
	Bribery is responsible for economic recession in Nigeria	IMP_6
	Bribery leads to misallocation of resources from critical sectors to non-productive sectors in Nigeria	IMP_7
	Bribery leads to low tax revenue which makes it impossible for governments to provide essentials services to ordinary people in Nigeria	IMP_8
	Bribery is responsible for high rate of poverty among ordinary people in Nigeria	IMP_9
	Bribery tarnishes the image of Nigeria in the international community	IMP_10
	Bribery discourages domestic investment in Nigeria	IMP_11
	Bribery discourages foreign investment in Nigeria	IMP_12
	Bribery transactions in the educational sector are responsible for falling standard of education in Nigeria	IMP_13
	Demand for bribes by government officials when employing workers means that qualified workers are never recruited in Nigeria	IMP_14

Table 2 shows that the gender of the respondents comprises of 66.5% male and 33.5% female. As indicated the majority of the respondents were male, this is because in Abuja majority of those who were willing to answer the questionnaire were male. The researcher and the research assistants tried to ensure that the questionnaires were administered evenly among the male and female gender, but some females refused to accept the questionnaire due to cultural and religious factors. In addition, about 55.3% of ordinary people engaged in economic activities in Nigeria are men [2]. The age of the respondents as indicated by Table 2 shows that those within the ages of 20 and below constitute 2.5%, 21 to 29 amounted to 20.3%, 30 to 39 were 35%, 40 to 49 stood at 29.8% while the remaining 11.8% were between the ages of 51 and above.

**Table 2:** Demographic characteristics of the respondents.

Variables Frequency Percentage		
Gender		
Male	556	66.5
Female	280	33.5
Age		
20 and Below	23	2.8
21- 29	170	20.3
30 −39	295	35.3
40 −49	249	29.8
50 and Above	99	11.8
Marital Status		
Single	247	29.5
Married	589	70.5
Family Size		
5 and Below	237	28.3
6 – 10	283	33.9
11 – 15	179	21.4
16 and Above	137	16.4
Educational Qualification		
No Formal Education	6	.7
Primary Certificate	7	.8
Secondary Certificate	22	2.6
Diploma/NCE	206	24.6
Table 4.7 continued		
Degree/HND	447	53.5
Master	133	15.9
PhD	15	1.8
Job Status		
Salaried Worker in the Private Sector	189	22.6
Civil Servant	537	64.2
Self-employed	(Farmers 9, petty traders 13,	
drivers 11, carpentry 7, market women 9,		
spare parts dealers 21, food vendors 36 and		
welders 4)	110	13.2
Annual Income		
N250, 000 ($685) and Below	108	12.9
N251, 000 – N350, 000 ($686 - $959)	42	5.0
N351, 000 – N450, 000 ($960 - $1233)	87	10.4
N451, 000 – N550, 000 ($1234 - $1507)	90	10.8
N551, 000 ($1508) and Above	509	60.9

In terms of marital status, Table 2 reveals that 70.5% of the respondents were married, while 29.5% were singles (including widows, divorcees and separated). This is because in Nigeria married individuals are expected to work and earn income in order to take care of their dependents. As such married individuals are more visible in offices, market places, and other commercial areas in Abuja, Nigeria. Table 2 also captures the family size of the respondents. It indicated that individuals with a family size of 5 and below constitute 28.3%, while that with 6 to 10 constitutes 33.9%, 11 to 15 have a family size of 21.4% and those with 16 and above accounted for 16.4%.

The educational status of the respondents was also shown in Table 2. It shows that 0.7% of the respondents had no formal education (0 years of schooling), 0.8% obtained primary school certificates (6 years of schooling), 2.6% have a secondary school certificates (12 years of schooling), 24.6% were diploma/NCE holders (14 years of schooling), 53.5% obtained degree/HND certificates (16 years of schooling), and 15.9% were having a master degree (18 years of schooling) while 1.8% possessed Ph.D. degrees (20 years of schooling). This is an indication that the majority of the respondents (71.2%) have higher educational qualifications (degree/HND, master and Ph.D.). This is likely because Abuja is a cosmopolitan city in which educated people are employed as public and private sector workers.

Statistically, Table 2 also presents the job status of the respondents. It indicates that 22.6% were salaried workers in the private sector 64.2% were civil servants and 13.2% were self-employed (which comprises Farmers, petty traders, drivers, carpenters, market women, spare parts dealers, food vendors and welders). The civil servants constitute the majority of those whom the questionnaires were administered because they are easily accessible. The annual income earned by the respondents was also captured by Table 4.7. The table reveals that 12.9% earned $685 and below, 5.0% earns $686 to $959, those who earned $960 to $1233 constitutes 10,4%, 10.8% had an annual income between $1234 to $1507 and 60.9% received $1508 and above as their annual income.

The issue of multicollinearity in the data was measured using tolerance (TV) and variance inflation factor (VIF) in SPSS version 23. The result is presented in Table 3. The table indicates that no variable included in the data violated the rules of thumb for both tolerance and VIF [3]. This implies that no two variables measured the same thing or phenomenon in the data. In other words, there is no severity of multicollinearity in the data.

**Table 3:** Results of multicollinearity test among the explanatory variables.

Variables	TV	VIF
SAC	0.784	1.276
AMB	0.955	1.047
IMP	0.783	1.277
GND	0.976	1.025
MTS	0.853	1.172
FAS	0.971	1.029
EDU	0.905	1.105
JOB	0.803	1.245

Note: Dependent variable = persistence of bribery (PBR); explanatory variables = societal acceptance of bribery (SAC), anti-bribery measures (ABM), socio-economic impacts of bribery (IMP), gender (GND), marital status (MTS), family size (FAS), educational qualification (EDU) and job status (JOB).

The reliability and validity of the questionnaire items in respect of their corresponding constructs were thoroughly investigated using partial least squares-structural equation modeling (PLS-SEM) as recommended [4]. In the PLS-SEM method reliability is tested using indicator and internal consistency reliability, whereas validity is tested using convergent and discriminant validity. Indicator reliability is determined by the outcome of factor loadings of a construct [5]. Internal consistency of a construct is determined by the value of Cronbach's alpha and Composite reliability [6]. Convergent and discriminant validity are determined by average variance extracted (AVE) statistics put forward by Fornell and Larcker [7].

Table 4 shows result of indicator reliability, composite reliability and convergence validity after 20 indicators out of 50 items included in the questionnaire were deleted due to low loadings and in order to improve the composite reliability. Table 4 shows that based on factor loadings, composite reliability and AVE the constructs and items demonstrates reliability, consistency and convergence in the present data.

**Table 4:** Indicator reliability, composite reliability and convergence validity.

Constructs/Indicators	Factor	Composite	AVE	Cronbach's
Loadings	Reliability	Alpha		
Anti-Bribery Measures	(ABM)	0.902	0.568	0.876
ABM2	0.734			
ABM3	0.770			
ABM4	0.738			
ABM9	0.680			
ABM10	0.778			
ABM11	0.789			
ABM12	0.734			
GND	(1.000)	(1.000)	(1.000)	(1.000)
EDU	(1.000)	(1.000)	(1.000)	(1.000)
FAS	(1.000)	(1.000)	(1.000)	(1.000)
JOB	(1.000)	(1.000)	(1.000)	(1.000)
MTS	(1.000)	(1.000)	(1.000)	(1.000)
Persistence of Bribery (PBR)	0.875	0.539	0.828	
PBR3	0.659			
PBR4	0.692			
PBR6	O.741			
PBR7	0.784			
PBR8	0.780			
PBR9	0.740			
Societal Acceptance (SAC)	0.846	0.525	0.772	
SAC1	0.694			
SAC3	0.713			
SAC4	0.788			
SAC5	0.775			
SAC8	0.643			
Socio-Economic Impacts (IMP)	0.887	0.529	0.851	
IMP4	0.673			
IMP8	0.681			
IMP9	0.711			
IMP10	0.794			
IMP11	0.784			
IMP12	0.739			
IMP13	0.698			

Note: Constructs with single items in parentheses.

Table 5 depicts the discriminant validity test based on [7]. The table shows that each of the latent construct's AVE is higher than its highest correlation with other latent constructs in the dataset. This is shown by the bolded AVE values 0.754, 0.734, 0.724 and 0.727, for ABM, PBR, SAC and IMP, respectively. This is an indication that each of the construct has achieved discriminant validity in the dataset.

**Table 5:** Discriminant validity: Fornell and Larcker Test.

Constructs	ABM	EDU	FAS	GND	JOB	MTS	PBR	SAC	IMP
ABM	**0.754**								
EDU	−0.086	(1.000)							
FAS	0.085	−0.132	(1.000)						
GND	−0.016	0.081	0.040	(1.000)					
JOB	0.101	0.227	0.001	0.052	(1.000)				
MTS	0.059	0.064	0.042	−0.087	0.359	(1.000)			
PBR	−0.103	0.049	0.016	0.049	−0.078	−0.028	**0.734**		
SAC	−0.131	−0.091	0.008	−0.035	−0.092	−0.066	0.552	**0.724**	
IMP	0.052	−0.033	0.022	0.024	−0.079	0.033	0.471	0.407	**0.727**

Note: Constructs with single items in parentheses.

## 2 Experimental Design, Materials and Methods

The data were acquired using a multiple stage sampling technique. The sampling was carried out in four phases (stages) and it involves clusters and simple random sampling methods. The first phase of the sampling involves dividing Abuja into six districts (clusters) namely; Abaji, Central Business, Bwari, Gwagwalada, Kuje and Kwali districts.

At the second stage, two districts were selected by simple random sampling from the six districts (the names of the six districts were written on six separate sheets of papers, then folded and thrown into a box, then mixed up, then two papers were picked randomly out of the six papers) Through these process Central Business and Bwari Districts were selected.

At the third stage, for each of the two districts selected at the second stage (Central Business District that has seven area councils and Bwari District that has ten area councils), three (3) area councils were selected also by a simple random sampling method as described above. In the Central Business district, Garki, Maitama and Wuse areas were selected. In Bwari District, Dutsen Alhaji, Kubwa and Katampe areas were selected. The six areas selected became the sampling frame.

The fourth stage involves self-administering of 1000 questionnaires by the researcher and six research assistants. The target participants were identified by the researchers using heads of human resource departments in public and private institutions, whereas officials of associations of self-employed individuals were used to identify self-employed respondents. An introductory letter issued by the researcher's institution was provided to officials of the targeted participants as evidence for data collection purposes.

The participants that were identified by the officials were randomly selected and handed the questionnaire. The participants were given two days to complete the questionnaire. In Garki, Maitama, Dutsen Alhaji, Kubwa and Katampe 140 questionnaires each were administered respectively while 160 were administered in Wuse to the grassroots.

The data collected from the respondents totaling 887 were screened for missing values and outliers in SPSS version 23, 836 responses were found to be useful for data analyses. The SmartPLS3.0 was used to analyze the useful data.

## Ethics Statement

The authors have obtained informed consent from the participants after assurances were given to the participants that no one will be identified in this data collection exercise (see supplementary material 1).

## CRediT Author Statement

**Abubakar Saddiq Sani:** Definition, Conceptualization, Investigation, Writing-original draft, Investigation, Formal analysis and Visualization. **Abu sufian Abu bakar:** Methodology, Software, Validation, Writing-Review and Editing and Supervision

## Declaration of Competing Interest

The authors hereby declared that they have no known competing interest financial or personal that could have influenced the outcome of this article.

## Appendix Supplementary materials

Image, application 1
