# Supplementary material for: Dataset on the perceptions of ordinary people on the persistence of bribery practices in Nigeria
Source: Data Brief. 2020 Dec 8;34:106616. doi: 10.1016/j.dib.2020.106616 (PMC7744688; doi:10.1016/j.dib.2020.106616)
Supplement: Supplementary file 1 [file mmc1.docx]

**Questionnaire**

**Ref No**

|  |  |  |
| --- | --- | --- |


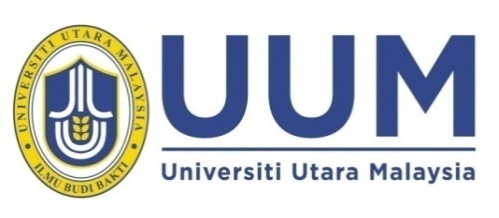


**School of Economics, Finance, and Banking,**

**Universiti Utara Malaysia, 06010 UUM Sintok,**

**Kedah Darul Aman,**

**Malaysia.**

Dear respected respondent, I am a Ph.D. student of the above-named institution undertaking a research on a topic titled Analysis of Persistence of bribery in Nigeria: Grassroots perspectives kindly give your opinion on the topic. All responses will be kept anonymous and no one will be identified in the research.

Please tick appropriate column as it applies to your opinion on each item in the questionnaire

**SECTION A: Demographic information**

|  | Question | Options | Response | |
| --- | --- | --- | --- | --- |
|  | Gender (GND) | [ ] Male  [ ] Female | \|  \| \| --- \| | |
|  | Age (AGE) | \|  \|  \|  \| \| --- \| --- \| --- \| | | |
|  | Marital Status (MTS) | [ ] Married  [ ] Single | | \|  \|  \| \| --- \| --- \| |
|  | Family Size (FAS) |  | | |
|  | Educational Qualification (EDU) | [ ] No formal education  [ ] Primary education  [ ] Senior secondary education (SSE)  [ ] Diploma/NCE  [ ] BSC/HND  [ ] Master  [ ] PhD | | \|  \| \| --- \| |
|  | Job Status (JOB) | [ ] Salaried employment in the private sector  [ ] Civil servant  [ ] Self-employed (Please Specify)............................... | | \|  \| \| --- \| |
|  | Please what is your annual Income? (ANY) | ~~N~~ | | |

**SECTION B. PERSISTENCE OF BRIBERY IN NIGERIA (PBR)**

**What is your opinion about the following statement? Do you strongly disagree, Disagree, Undecided, Agree or strongly agree?**

| **Labels/Codes** | **Statement** | **Strongly disagree** | **Disagree** | **Undecided** | **Agree** | **Strongly agree** |
| --- | --- | --- | --- | --- | --- | --- |
| PBR_1 | Bribery is widespread in Nigeria. | 1 | 2 | 3 | 4 | 5 |
| PBR_2 | Bribery has become permanent in Nigeria. | 1 | 2 | 3 | 4 | 5 |
| PBR_3 | Bribery has continued to occur in Nigeria despite combative policy measures put in place by the government. | 1 | 2 | 3 | 4 | 5 |
| PBR_4 | Bribe is frequently offered to school officials in order to get services needed from educational institutions in Nigeria. | 1 | 2 | 3 | 4 | 5 |
| PBR_5 | Bribe is frequently offered to medical officers in order to access medical services in Nigeria. | 1 | 2 | 3 | 4 | 5 |
| PBR_6 | Bribe is frequently offered to government officials in order to get official documents (such as birth certificate, tax clearance, national identity card, driver’s licence, voter’ card and certificate of occupancy among others) in Nigeria. | 1 | 2 | 3 | 4 | 5 |
| PBR_7 | Bribe is frequently offered to police officers in order to get their assistance or to avoid paying a fine or arrest in Nigeria. | 1 | 2 | 3 | 4 | 5 |
| PBR_8 | Bribe is frequently offered to court officials in order to get their assistance or to have judgement in one’s favour in Nigeria. | 1 | 2 | 3 | 4 | 5 |
| PBR_9 | Bribe is frequently offered in order to secure a job in Nigeria. | 1 | 2 | 3 | 4 | 5 |
| PBR_10 | Bribery is the most important problem facing Nigeria today. | 1 | 2 | 3 | 4 | 5 |

**SECTION C SOCIETAL ACCEPTANCE OF BRIBERY (SAC)**

**What is your opinion about the following statement? Do you strongly disagree, Disagree, Undecided, Agree or strongly agree?**

| **Labels/Codes** | **Statement** | **Strongly disagree** | **Disagree** | **Undecided** | **Agree** | **Strongly agree** |
| --- | --- | --- | --- | --- | --- | --- |
| SAC_1 | Bribery has become a way of life in Nigeria. | 1 | 2 | 3 | 4 | 5 |
| SAC_2 | Bribery is justifiable in Nigeria. | 1 | 2 | 3 | 4 | 5 |
| SAC_3 | Bribery is tolerated in Nigeria. | 1 | 2 | 3 | 4 | 5 |
| SAC_4 | Ordinary government officials asking for bribes openly in Nigeria. | 1 | 2 | 3 | 4 | 5 |
| SAC_5 | Ordinary people offering bribes openly in Nigeria. | 1 | 2 | 3 | 4 | 5 |
| SAC_6 | Ordinary people do not report bribery offences to anti-bribery institutions in Nigeria. | 1 | 2 | 3 | 4 | 5 |
|  |  |  |  |  |  |  |
| SAC_7 | Ordinary people have a non-charlatan attitude towards bribery in Nigeria. | 1 | 2 | 3 | 4 | 5 |
| SAC_8 | Ordinary people aiding bribery transactions in Nigeria. | 1 | 2 | 3 | 4 | 5 |
| SAC_9 | Everyone is involved in bribery in Nigeria. | 1 | 2 | 3 | 4 | 5 |

**SECTION D ANTI-BRIBERY MEASURES (ABM)**

**What is your opinion about the following statement? Do you strongly disagree, Disagree, Undecided, Agree or strongly agree?**

| **Labels/Codes** | **Statement** | **Strongly disagree** | **Disagree** | **Undecided** | **Agree** | **Strongly agree** |
| --- | --- | --- | --- | --- | --- | --- |
| ABM_1 | Establishment of Code of Conduct Bureau (CCB) has reduced persistence of bribery in Nigeria. | 1 | 2 | 3 | 4 | 5 |
| ABM_2 | Establishment of Public Complaints Commission (PCC) has reduced persistence of bribery in Nigeria. | 1 | 2 | 3 | 4 | 5 |
| ABM_3 | Establishment of National Orientation Agency (NOA) has reduced persistence of bribery in Nigeria. | 1 | 2 | 3 | 4 | 5 |
| ABM_4 | War Against Indiscipline and Corruption (WAIC) has reduced persistence of bribery in Nigeria. | 1 | 2 | 3 | 4 | 5 |
| ABM_5 | Establishment of Independent Corrupt Practices Related Offences Commission (ICPC) has reduced persistence of bribery in Nigeria. | 1 | 2 | 3 | 4 | 5 |
| ABM_6 | Establishment of Economic and Financial Crimes Commission (EFCC) has reduced persistence of bribery in Nigeria. | 1 | 2 | 3 | 4 | 5 |
| ABM_7 | Prosecution and conviction of offenders has reduced persistence of bribery in Nigeria. | 1 | 2 | 3 | 4 | 5 |
| ABM_8 | Whistle blowing policy has reduced persistence of bribery in Nigeria. | 1 | 2 | 3 | 4 | 5 |
| ABM_9 | Deterrence measures (such as enhancing integrity and transparency) have reduced persistence of bribery in Nigeria. | 1 | 2 | 3 | 4 | 5 |
| ABM_10 | Citizen advocacy measures have reduced persistence of bribery in Nigeria. | 1 | 2 | 3 | 4 | 5 |
| ABM_11 | Civil Society anti-bribery campaigns have reduced persistence of bribery in Nigeria. | 1 | 2 | 3 | 4 | 5 |
| ABM_12 | Targeting high profile bribery offenders has reduced persistence of bribery in Nigeria. | 1 | 2 | 3 | 4 | 5 |

**SECTION E SOCIO-ECONOMIC IMPACTS OF BRIBERY IN NIGERIA (IMP)**

**What is your opinion about the following statement? Do you strongly disagree, Disagree, Undecided, Agree or strongly agree?**

| **Labels/Codes** | **Statement** | **Strongly disagree** | **Disagree** | **Undecided** | **Agree** | **Strongly agree** |
| --- | --- | --- | --- | --- | --- | --- |
| IMP_1 | When bribery is offered by ordinary people in Nigeria it is like a regressive tax. | 1 | 2 | 3 | 4 | 5 |
| 1MP_2 | Bribery discourages ordinary people from assessing public goods in Nigeria. | 1 | 2 | 3 | 4 | 5 |
| IMP_3 | Bribery reduces the social welfare of ordinary people in Nigeria. | 1 | 2 | 3 | 4 | 5 |
| IMP_4 | Bribery is responsible for erosion of ordinary people’s trust in private and government institutions in Nigeria. | 1 | 2 | 3 | 4 | 5 |
| IMP_5 | Bribery raises the cost of doing business in Nigeria. | 1 | 2 | 3 | 4 | 5 |
| IMP_6 | Bribery is responsible for economic recession in Nigeria. | 1 | 2 | 3 | 4 | 5 |
| IMP_7 | Bribery leads to misallocation of resources from critical sectors to non-productive sectors in Nigeria. | 1 | 2 | 3 | 4 | 5 |
| IMP_8 | Bribery leads to low tax revenue which makes it impossible for governments to provide essentials services to ordinary people in Nigeria. | 1 | 2 | 3 | 4 | 5 |
| IMP_9 | Bribery is responsible for high rate of poverty among ordinary people in Nigeria. | 1 | 2 | 3 | 4 | 5 |
| IMP_10 | Bribery tarnishes the image of Nigeria in the international community. | 1 | 2 | 3 | 4 | 5 |
| IMP_11 | Bribery discourages domestic investment in Nigeria. | 1 | 2 | 3 | 4 | 5 |
| IMP_12 | Bribery discourages foreign investment in Nigeria | 1 | 2 | 3 | 4 | 5 |
| IMP_13 | Bribery transactions in the educational sector are responsible for falling standard of education in Nigeria. | 1 | 2 | 3 | 4 | 5 |
| IMP_14 | Demand for bribes by government officials when employing workers means that qualified workers are never recruited in Nigeria. | 1 | 2 | 3 | 4 | 5 |

**Thank you for participating in this data collection.**
